# Designing a regional clinical service for people with early‐onset type 2 diabetes in England

**DOI:** 10.1111/dme.15479

**Published:** 2024-11-25

**Authors:** Jonathan Goldney, Victoria Alabraba, Priscilla Sarkar, Harriet Morgan, Malak Hamza, Michael Skarlatos, Tommy Slater, Jack A. Sargeant, Rhys O'Callaghan, Michelle Hadjiconstantinou, Julia Burdon, Azhar Farooqi, Samuel Seidu, Claire Meek, Melanie J. Davies

**Affiliations:** ^1^ Diabetes Research Centre University of Leicester Leicester UK; ^2^ National Institute for Health Research (NIHR) Leicester Biomedical Research Centre Leicester UK; ^3^ Leicester Diabetes Centre University Hospitals of Leicester NHS Trust Leicester UK; ^4^ Clinical Research Network (CRN) East Midlands, NIHR University Hospitals of Leicester NHS Trust Leicester UK

**Keywords:** challenges, clinical service, early onset, primary care, secondary care, service design, type 2 diabetes mellitus

## Abstract

**Aims:**

To design a regional clinical service for people with early‐onset type 2 diabetes (EOT2D) in Leicester, Leicestershire and Rutland (England).

**Methods:**

A literature search was undertaken to identify important considerations. A working group of key stakeholders was formed to design a triage system and service pathway. Electronic medical records (EMRs) were searched (15th November 2023) to assess feasibility of the pathway and adapt accordingly.

**Results:**

A literature search identified important considerations: High risk of complications; large proportion from minority ethnic and socioeconomically deprived backgrounds; significant psychological burden; stigma and other social challenges; and misclassification and miscoding. Novel clinical risk criteria were developed, implementable in EMRs, to match intervention‐intensity to clinical need. Specialist clinics were planned, one for people at the highest‐clinical risk, another for women with adverse perinatal risk factors. A healthcare professional training package was developed to increase awareness of the unmet clinical needs of people with EOT2D and to upskill in provision of holistic care. Subsequent EMR searches supported the need for our service. Due to the large numbers with HbA1c ≥86mmol/mol (10.0%; n=299; 10.8% of total), these people were prioritised for clinic access. We opted for specialist nurse/educator support to practices with clustering of patients and to financially incentivise referrals from primary care into services.

**Conclusions:**

We showcase a service specifically for people with EOT2D based on the literature, a broad range of stakeholder involvement and utilising a locally‐sourced data‐driven approach. We further discuss areas for development and recommendations based on the challenges we encountered.

**What's new?**

- There is a national drive to improve services for people with early‐onset type 2 diabetes (EOT2D) including provision of funding to local regions, however, a lack of published examples to guide best practice.
- From the literature, we summarise important factors to consider in service design, describe how this was utilised to design our service, and undertake searches of electronic medical records to understand pathway feasibility.
- To our knowledge this is the first paper to describe this process, with the aim of aiding future service design by showcasing our practice and sharing recommendations from the lessons learned and challenges faced.

## 1 BACKGROUND

The prevalence of early‐onset type 2 diabetes (EOT2D; diagnosis aged <40 years) is increasing.^1^ EOT2D has a distinct phenotype from later‐onset T2D, with an increased risk of complications^2^; however, there is little research into effective interventions for this group specifically.

The area of EOT2D has accrued much interest nationally in the UK. This has, in part, been driven by the National Diabetes Audit (NDA), an audit of 97% of people with EOT2D in England and Wales which monitors care provision and outcomes.^3^, ^4^ The increasing prevalence of EOT2D, also observed within the NDA, led to a specific report on the characteristics and care of people with EOT2D.^4^ This report revealed that people with EOT2D are receiving suboptimal care, with only 34% of people with EOT2D aged 26–39 years receiving all nine national care processes (a national initiative to improve diabetes monitoring and management). Notably, only 55% were achieving an HbA1c 58 mmol/mol (<7.5%), compared to 68% of 60–79‐year‐olds.

As a result, national funding has become available for two‐years (April 2023–March 2025) to all Integrated Care Boards (ICBs; the regional commissioners for health services in England) to provide extra care for people with EOT2D currently aged <40 years under the T2Day programme.^5^ The programme has three broad aims: (1) to provide high quality care and optimise glycaemia, cardiovascular risk and weight, aiming to reduce long‐term complications (2) to support better preparation for pregnancy in women; (3) to help address unmet psychological and social needs and support overall well‐being. Alongside treatment targets, the programme provides suggested ways to utilise funds, with funding modelled on a 30 minute clinical review per patient within the locality (£52 per patient per year); however, local implementation can be flexibly determined by ICBs allowing for services to be adapted to local need.

Despite this national drive to improve services, there is a lack of literature showcasing how services for people with EOT2D have been developed. In this paper, we present the breadth of the EOT2D service development in Leicester, Leicestershire and Rutland (LLR) ICB thus far within the confines of the T2Day funding, to share practice and raise challenges for future consideration. This includes designing risk stratification criteria for triage purposes, a ‘high‐risk’ clinic for people with the highest‐clinical need, a pre‐conception clinic for women planning pregnancy or at risk of pregnancy‐related complications, a healthcare professional (HCP) training package to facilitate regional upskilling, and referral into relevant clinical trials.

## 2 OVERARCHING STRATEGY

Our pathway development comprised 3 sequential phases of work:

*Phase 1*: A literature search was undertaken to identify important considerations when designing an EOT2D‐specific service.
*Phase 2*: A regional pathway was designed for the management of people with EOT2D.
*Phase 3*: Electronic medical records (EMRs) were searched to assess feasibility of the pathway.

## 3 PHASE 1: IDENTIFY IMPORTANT FACTORS TO CONSIDER IN SERVICE DESIGN

From the literature, we identified several important themes of factors to consider in designing a service for people with EOT2D: A population at risk of complications; a different patient demography with earlier versus later‐onset of T2D; adverse psychological factors; stigma and other social factors; frequent misclassification and miscoding in EMRs.

### 3.1 A population at risk

People with EOT2D represent a population at risk of poor outcomes. A younger age at diagnosis results in a greater exposure to hyperglycaemia, both through a longer duration of diabetes, and higher glucose throughout the disease course as compared to T2D diagnosed later in life.^6^ Further to this, people with EOT2D have a more severe cardiovascular risk phenotype compared to later‐onset diabetes, including a higher body mass index (BMI), waist circumference and more adverse lipid profile.^7^ This culminates in a greater risk of microvascular and macrovascular outcomes in people with EOT2D compared to later‐onset disease and increased risk of mortality.^2^, ^8^

There is little data on the efficacy of interventions tested in people with EOT2D specifically, despite the increased‐risk phenotype. Indeed, guidelines that cover all adults with T2D are largely developed from trials that exclude patients with EOT2D,^9^ and trials in younger individuals are mostly specific to adolescent populations.^1^ The higher BMI associated with EOT2D is emerging as a potential important focus of pharmacological treatment. Despite a lack of data generally, tirzepatide may have particular efficacy in young adults, with a mean reduction in HbA1c of 2.6% (approximately 29 mmol/mol) and body weight of 14 kg at 40 weeks in a post hoc analysis of the SURPASS‐2 randomised controlled trial, similar to the effects in older adults, although long‐term outcomes are lacking.^10^

Women with EOT2D also have increased risk of pregnancy‐related complications. From 260 pregnancies of women diagnosed as adolescents in the US, 25.3% experienced pregnancy loss.^11^ Additionally, 17.9% of offspring were in the macrosomic range (>4 kg), 10.2% had cardiac anomalies and 10.3% had other congenital anomalies. In the UK, stillbirths are similarly high amongst women with EOT2D, affecting 13.5 per 1000 pregnancies,^12^ with only 22.2% on folic acid supplementation and many women taking teratogenic medications at conception. Furthermore, there are more pregnant women with T2D than type 1 diabetes (T1D), and women with T2D also present two weeks later on average.^12^ The long‐term implications of teratogenic medications, complications and traumatic experiences are unknown for both the future health of the mother and offspring.

Infertility/subfertility may also be an issue as T2D may adversely affect the neuroendocrine control of reproduction.^13^ T2D is likely a causative factor for erectile dysfunction and infertility in men,^14^ whilst women are at higher risk of polycystic ovary syndrome and subsequent subfertility.^15^

### 3.2 Demographic characteristics of those diagnosed <40 years

In the UK, people with EOT2D are more likely to be from minority ethnic groups and live in the most‐deprived socioeconomic areas as compared with people diagnosed later in life.^4^ This is worrying as deprivation is a stronger risk factor for suboptimal management of diabetes than age, type and duration of diabetes, and BMI.^16^ Similarly, adolescents from minority ethnic backgrounds are at higher risk of complications.^17^ People with EOT2D diagnosed aged <25 years are also more likely to be women, whereas the proportion of men and women diagnosed at ages 26–39 years is similar.^4^

### 3.3 Psychological factors

As well as physical complications, EOT2D is associated with a significant psychological burden. Indeed, rates of hospitalisation related to mental illness are higher in those with EOT2D compared to later‐onset,^18^ as is the prevalence and severity of depressive symptoms.^19^ Moderate‐to‐high levels of diabetes‐specific distress are more prevalent in adults with EOT2D than in those with later‐onset: 50.0% in EOT2D versus 19.3% diagnosed aged >60 years.^19^ People with EOT2D also report higher levels of self‐judgment and isolation, and lower overall self‐compassion.^19^ In turn, depression and distress have been associated with poorer quality of life, suboptimal glycaemic outcomes and diabetes complications.^20^

People with EOT2D and psychiatric comorbidities taking atypical antipsychotic medications may represent an under‐recognised cohort at high risk, due to associated weight gain and reduced insulin sensitivity in a dose‐dependent manner.^21^ Similarly, eating disorders may affect 10–40% of people with T2D and are most prevalent in early adulthood.^22^ This is perhaps due to the additional attention given to diet, the psychological pressure of the diagnosis, coexisting depression and body dysmorphia.^22^ Disordered eating may also be a risk factor for EOT2D.^22^ Disordered eating behaviours, particularly binge‐eating and night‐time eating, can lead to weight gain, suboptimal glycaemia and negatively impact mental health.^22^

### 3.4 Stigma and other social factors

Many individuals with EOT2D are affected strongly by negative social judgement, known as diabetes stigma.^23^ Whilst this area is generally under‐researched,^23^ it's clear that the burden of stigma is extremely high in people with EOT2D.^24^, ^25^, ^26^ Fear of judgement, or negative social repercussions, from disclosure of diabetes is a key theme identified in several qualitative studies.^24^, ^25^, ^26^ This has led individuals to feel shame, self‐blame and embarrassment as well as exclusion from society.^25^ A younger age at diabetes diagnosis may be an exacerbating factor, as stigmatisation has been exacerbated for individuals by perceptions that T2D is a disease of older age as well as experiencing healthcare services which are designed for older people.^26^ Indeed, HCPs and organisations can be key drivers of diabetes stigma,^23^ with people with EOT2D describing experiences such as judgment by HCPs.^25^

There are other key social factors to consider. Individuals with EOT2D often have a strong family history of T2D, being present in 84% of adolescents.^27^ Families may share similar health beliefs, lifestyles, cultural beliefs, religious values and environments that may increase risk, especially in cases of multigenerational living in the same household.^28^ As discussed, there is a higher prevalence of EOT2D in people from minority ethnic groups, where sociocultural pressures in families and communities may affect eating habits and adherence to dietary guidelines.^28^, ^29^ Younger, unmarried individuals from South Asian communities especially struggle with stigma; while positive family support has conversely been reported to be beneficial in married individuals in these communities.^28^

Engagement with healthcare services can also be challenging for adults with EOT2D due to employment pressures in early careers, lack of flexibility in less‐skilled or unstable employment, being in full‐time education and/or having young families. This can have a negative psychological impact and may affect self‐management of diabetes, for example meal‐planning and ability to take medications as advised.^1^ Language barriers can also affect access to healthcare and health literacy in ethnic minority groups.^28^ These challenges may be heightened by higher levels of socioeconomic deprivation.

### 3.5 Misclassification and miscoding

Misclassification of diabetes type is a common issue, affecting as many as 27.2% and 18.9% of people with T1D and T2D, respectively.^30^ This issue may be more common in young people, particularly given the similar prevalence of T1D and T2D in early adulthood, and peak incidence of maturity‐onset diabetes of the young (MODY). Miscoding (assigning a vague diagnostic code) is even more prevalent: 47.8% in a regional UK study,^30^ with misdiagnosis in 6.1%.

## 4 PHASE 2: DESIGNING A REGIONAL PATHWAY

### 4.1 Risk stratification to match intervention‐intensity to clinical need

Some people with EOT2D are at a higher risk of complications than others, requiring a more intensive and more costly intervention than people at lower risk. Because currently no criteria exist, we first established a working group to define risk criteria to allow triage into different interventions. The working group included clinicians from both primary and secondary care, academics and senior figures from the ICB and local NHS Trust. The working group was chaired by a member of the ICB, and multiple iterative versions of the risk criteria were discussed and drafted until members agreed unanimously with the final version. Whilst the risks associated with EOT2D are clear, there is little data to guide who within this group may be at higher versus lower risk of complications; therefore, we were largely reliant on evidence from other populations, and our local expert experience and opinion. Generally, we wanted criteria to avoid an overly glucocentric approach, recognising the importance of other factors (e.g. dyslipidaemia, obesity and hypertension). We also aimed to identify risk factors for non‐vascular complications, namely psychological and psychiatric burden, and pregnancy‐related complications. Furthermore, it was important that parameters used to triage were limited to those routinely collected, to allow implementation in EMRs and facilitate a quick and cost‐effective process.

**FIGURE 1:**
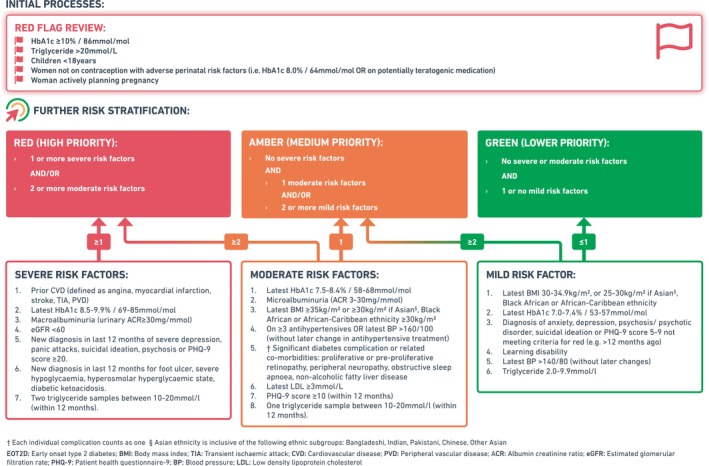
Leicester early‐onset type 2 diabetes risk stratification criteria.

The criteria shown in Figure 1 were agreed, including Red, Amber and Green (RAG) categories. These came from three lists of factors, which we felt were indicative of three different levels of risk. To account for these factors potentially being additive, RAG categories were created dependent on the number and severity of risk factors present. People in the ‘high‐risk’ red category would receive the highest level of intervention – for example, review(s) within a specialist setting. The ‘medium‐risk’ amber category involved lower‐priority high‐level intervention, or for a less resource‐intensive intervention, and the ‘low‐risk’ green category for simple or low‐priority intervention. Additionally, we wanted to identify people at the highest risk of complications for ‘Red flag review’. HbA1c ≥86 mmol/mol (10.0%) was used to identify these people, due to it being a strong predictor of mortality and complications,^31^ and a good surrogate for poor access to care. High triglyceride (>20 mmol/L) was also used to identify people for ‘red flag review’ due to the associated risk of acute pancreatitis. Similarly, children aged <18 years should be managed by a specialist multidisciplinary team. We also aimed to quickly review women actively planning pregnancy, and those not on contraception with adverse perinatal risk factors (HbA1c ≥64 mmol/mol/ 8.0% or on potentially teratogenic medication) to prevent pregnancy‐related complications.

### 4.2 Pathway to identify and triage people within the region

**FIGURE 2:**
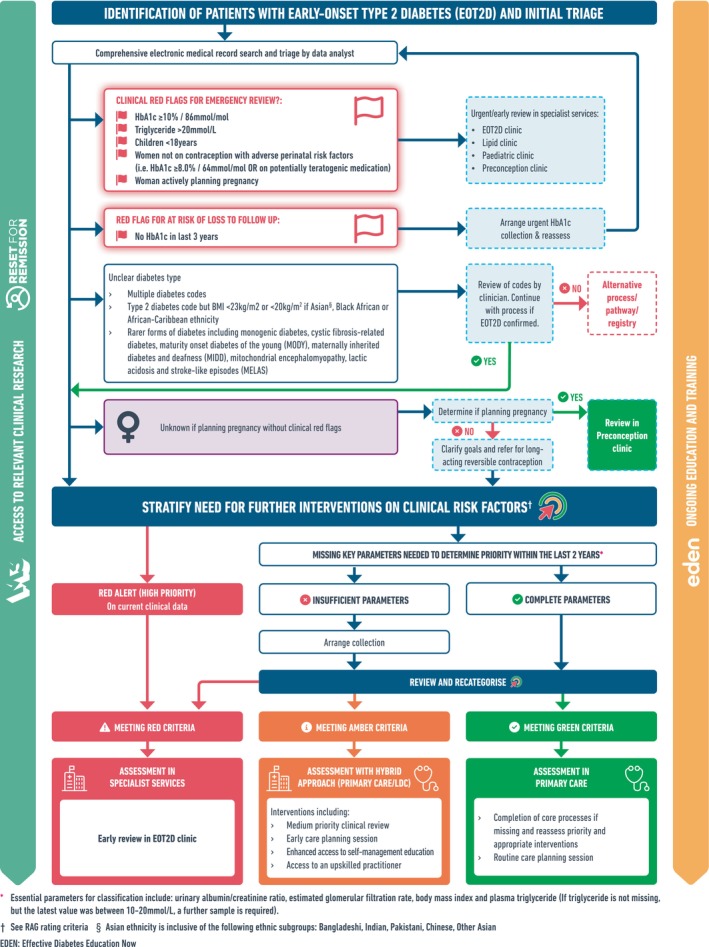
Leicester early‐onset type 2 diabetes clinical service pathway.

Utilising lessons learned from the literature (shown in Table 1), alongside the developed risk stratification criteria, a service pathway was drafted (shown in Figure 2). In this pathway, people at immediate risk (i.e. Red flag review), were foremostly prioritised to be seen in a new specialist bespoke MDT clinic (consultant diabetologist, registrar, specialist nurse) to conduct a comprehensive assessment and holistic care plan with a view to either (a) discharge back to primary care with a long‐term care plan, (b) enrol in a relevant research trial, (c) continue management within a secondary‐care service. Furthermore, despite children being out of scope of the T2Day programme,^5^ they could be easily triaged into pre‐existing paediatric clinics, and so were incorporated in the pathway. Given the clear needs for women planning pregnancy to be better prepared, those actively planning, or those with perinatal risk factors, would be triaged to a bespoke pre‐conception clinic. Primary care practices would be provided with pre‐made EMR searches that they could easily undertake to identify all patients that were eligible for these specialist clinics, and encouraged to refer to services.

**TABLE 1:** Important factors identified from the literature and subsequent adaptations to the service pathway.

Factor	Pathway adaptations
High risk of diabetes complications (macrovascular, microvascular, psychiatric, fertility‐ or pregnancy‐related) and reduced life expectancy	Inclusion of a bespoke specialist clinic, targeting those at highest risk initially (HbA1c ≥10% and/or triglycerides ≥20 mmol/L). Intensive cardiovascular risk factor management is recognised as a cornerstone of management, alongside management of psychiatric comorbidity Development of RAG criteria to determine risk of complications including a wide range of clinical parameters Inclusion of a novel pre‐conception clinic A module built into HCP education package that highlights these factors
A lack of data for effective interventions and pharmacotherapies, with some data supporting the efficacy of tirzepatide as a potent glucose‐ and weight‐ lowering agent A large proportion of pregnant women on teratogenic medication	Consideration of tirzepatide in people with raised glucose and weight To closely follow guidelines for intensification of medication for other complications such as hypertension and dyslipidaemia To caution women about the teratogenicity of medications and recommend concurrent contraception in clinics To discuss treatment options in pre‐pregnancy clinic for women planning pregnancy
Low attainment of national diabetes targets likely to result in EMRs with missing clinical data	Extra assistance to practices with high numbers of patients who may be struggling with access to healthcare Communications with practices and sharing of searches to allow them to quickly identify patients with miscoding
A greater proportion of people from minority ethnic and more socioeconomically deprived backgrounds	Recognition that there may be language and/or cultural barriers that need to be accounted for Clinical services need to have reach into all communities and be acceptable to both men and women and from people of all cultures A need for evaluation of inclusion and reach
Psychological factors including diabetes distress, adverse eating behaviours and depression	A module built into HCP education highlighting these factors We recognise there may be future need to include more formal pathways for psychological/psychiatric management, such as access to a health psychologist or bespoke eating‐disorder services
Social factors including busy lives (young families, early careers, full‐time education), stigma, poor access to health care, inflexible working environments, strong family history and multigenerational history of diabetes	A module built into HCP education highlighting these factors Flexible booking for bespoke specialist clinic, different times of day available, flexibility for evening appointments as well as virtual/ telephone consultations available Extra assistance to practices with high numbers of patients who may be struggling with access to healthcare
Poor diagnostic coding	A HCP to review the patients’ clinical records to correct coding, or to review the patient with further investigation (e.g. c‐peptide/ autoantibodies) as required

Abbreviations: EMRs, electronic medical records; HCP, healthcare professional; RAG, Red‐Amber‐Green.

## 5 PHASE 3: ELECTRONIC MEDICAL RECORD SEARCH

Various searches of the EMRs for all people living in LLR were undertaken on 15th November 2023 to (a) assess the feasibility of our proposed pathway and refine it accordingly; (b) assess the utility of our RAG criteria by various thresholds of individual parameters to be able to adapt criteria based on local need; (c) assess clustering of people by stratifying results by practice. Consent and ethical approval was not required as work was undertaken for service improvement purposes.

A summary of search results is shown in Table 2. A total of 2772 adults (35.0% women) and 40 children with EOT2D were identified. Overall, the search results supported the need for our pathway. The need for a specialist clinic was also reaffirmed, particularly with 299 (10.8%) people with HbA1c ≥86 mmol/mol (10.0%) and 1188 (42.9%) people meeting Red criteria. Given the large number with high HbA1c, to provide the maximal benefit with the allocated resource, we planned to initially limit the specialist clinic to ‘Red flag’ criteria only and to then delimit when capacity allows. Additionally, 468 (48.2%) women were at high risk of a pregnancy‐related complication, affirming the need for a bespoke pre‐pregnancy clinic. As expected, there were also large issues with miscoding. For example, 319 people had a code of both T1D and T2D and 2384 people had only a generic diabetes code. Finally, we highlighted the large issue of missing data, meaning people could not be categorised into RAG criteria, for example 657 people (23.7%) could not be excluded from Red criteria due to missing key parameters. Of note, 29 participants (1.0%) had not had a HbA1c performed in the last 3 years, and 869 people (31.3%) had never had urinary albumin: creatinine ratio tested.

**TABLE 2:** Summary of search results.

Purpose of search	Search	Number
Totals	Number of patients with EOT2D^a^	2772
	Children with Type 2 diabetes	40
Miscoding (assigning a vague diagnostic code)	Both T1D and T2D codes	319
	Generic diabetes code without a further T1D or T2D code	2384
Red flag clinical variables	High HbA1c ≥10% (% of total)	299 (10.8%)
	Triglyceride ≥20 mmol/L (% of total)	7 (0.3%)
Women's health	Total number of women (% of total)	971 (35.0%)
	Women not on contraception (% of women)	842 (86.7%)
	Planning pregnancy (% of women)	17 (1.8%)
	At risk of pregnancy complication: HbA1c ≥64 mmol/mol (8.0%) OR on potentially teratogenic medication (% of women)	468 (48.2%)
Risk stratification	Red (% of total)	1188 (42.9%)
	Amber (% of total)	262 (9.5%)
	Green (% of total)	66 (2.4%)
Unable to categorise due to missing data	Not meeting Red criteria but missing key parameters^b^ (% of total)	657 (23.7%)
	Not meeting Amber or Red criteria but missing key parameters^b^ (% of total)	599 (21.6%)

^a^ Excludes those also with an additional code of T1D.

^b^ Key parameters included: urinary albumin: creatinine ratio, estimated glomerular filtration rate, body mass index, plasma triglyceride (if last triglyceride was 10‐20 mmol/L and had not been repeated since then this was also counted as missing).

We identified a clustering of practices where there was a large number of people with either confirmed EOT2D, miscoding (ambiguous diagnosis) and/or missing key parameters. Following this, we decided that a specialist educator/ diabetes specialist nurse, would contact these practices to offer to visit the site or work remotely to provide additional ad hoc healthcare support/upskilling, mentoring and facilitate the referral of eligible people into the developed service pathways. Given competing pressures on primary care, we also incentivised referrals into our specialist clinic and pre‐pregnancy clinic by reimbursement of £5 per referral for primary care practices, given previous relative success of pay‐for‐performance incentives.^32^

## 6 ADDITIONAL PLANNED INTERVENTIONS

Given that we foresaw potential need for improvement over several additional areas, such as with clinical coding (misdiagnosis), missing clinical data, and HCPs potentially inadvertently worsening diabetes stigma, we designed an online training package that could be rolled out in parallel to the service pathway. The aim was to increase the awareness of local HCPs to the unmet clinical needs of people with EOT2D, incorporating factors identified in Phase I (Table 1), and to upskill through discussion of important components of holistic care specific to this group. Effective Diabetes Education Now, an NHS education team based at Leicester Diabetes Centre,^33^ was commissioned to develop the online training programme. The developed HCP training package consisted of three modules encompassing: pre‐pregnancy care, cardio‐renal and metabolic management, psychological well‐being and person‐centred care (including discussion of diabetes stigma). The roll out of this package to optimise HCP reach is still being determined.

Throughout the pathway we planned to recruit people into ongoing local clinical trials (such as the RESET 4 REMISSION^34^ and M3 studies^35^), to facilitate access to novel and tailored interventions. This is particularly important for people with EOT2D, who are underrepresented in research trials.^9^

## 7 DISCUSSION

This paper summarises our regional approach to utilise the funds from the T2Day programme. We created a regional service for people with EOT2D with tiered interventions based on clinical need, alongside regional HCP upskilling. To our knowledge, this is the first paper to describe bespoke clinical services targeted specifically for the needs of people with EOT2D.

### 7.1 Future areas for development locally

This service pathway was developed within the financial constraints of the T2Day funding. We feel that there are still ways our service could be improved. Firstly, we did not design a specific component that addressed the psychological burden in our cohort, although it is featured in our HCP training package and we also plan to maximise the use of locally available psychological support referral pathways within the clinics. Further work is required to determine cost‐effective interventions to meet the psychological needs of people with EOT2D and how to design services to deliver these. Similarly, we are also planning further low‐cost interventions, namely provision of a digital self‐management programme, to provide care for people with EOT2D that do not meet eligibility for the specialist clinics, but this still under development.

Furthermore, forthcoming research may offer tools to improve local services. Whilst we identified from both the literature and our local data that misclassification and miscoding are prevalent issues for people with EOT2D; our work with HCP upskilling and specialist nurse supporting visits may not solve these issues. Whilst none are currently validated specifically for adults aged <40 years using routine clinical data, we envisage that prediction algorithms may prove to be more cost‐effective and accurate, utilising the strengths of EMRs. These algorithms may not be far away and could be used for targeted c‐peptide testing. Similarly, we also identified missing data as an issue, but our service does not feature a specific component to tackle this; there may also be tools developed that can support primary care in addressing this.

### 7.2 Recommendations

Our service needs to be implemented and evaluated to investigate whether it is associated with an improvement in clinical outcomes. There is a clear need for interventions to reduce the risk of complications in people with EOT2D; however, a lack of evidence on how best to achieve this.^9^ Ongoing and future research may provide evidence‐based interventions specific for people with EOT2D, but these will take years to materialise. In the meantime, initiatives like the T2Day Programme are vital, as a lack of intervention is likely to have significant implications on both public health and national finances, given the rising prevalence and increased morbidity of EOT2D in working‐age adults. Our service pathway enables sharing of ideas and evidence‐grounded expert opinion to hopefully allow immediate improvement of care in other regions in lieu of better‐quality evidence.

We also identified that the quality of EMRs, specifically the large amount of missing data, is likely to be a significant barrier to implementing low‐cost interventions utilising EMRs both locally and nationally. For example, many people do not have a specific diabetes diagnosis, meaning that we are likely to be missing people from our pathway, limiting the reach. The paucity of recorded data in records additionally limits the effectiveness of triage. Improvements in data accuracy and completeness are needed. Better support is needed for primary care to address this, alongside research into barriers to effective clinical coding and data collection. Specific interventions may also be helpful, such as HCP education and/or better utilisation of technology.

Another barrier is that due to restrictions on data sharing and consent, we would be unable to directly invite people into secondary‐care services from primary care. Similarly, to undertake the anonymised searches ourselves, multiple permissions were needed from local stakeholders, leading to delays. A streamlined consent and data sharing process is required to minimise both barriers to healthcare interventions and patient burden.

## 8 conclusion

We provide the first example of a regional service designed specifically for people with EOT2D. Our service was designed using current literature, expert opinion, a broad range of stakeholder involvement and a locally sourced data‐driven approach. We also discuss future areas for development and showcase methods that could improve care for people with EOT2D.

## FUNDING INFORMATION

The service design was funded by NHSE T2Day program by the LLR ICB. This work was supported by the National Institute of Health Research (NIHR) Leicester Biomedical Research Centre. JG is supported by the Wellcome Trust Leicestershire Healthcare Inequalities Improvement Doctoral Training Programme (223512/Z/21/Z). MJD is co‐funded by the NIHR Leicester Biomedical Research Centre and University of Leicester.

## CONFLICT OF INTEREST STATEMENT

Professor Melanie Davies has acted as consultant, advisory board member and speaker for Boehringer Ingelheim, Eli Lilly, Novo Nordisk and Sanofi, an advisory board member Pfizer, AstraZeneca, Zealand Pharma, Carmot/Roche, Amgen and Medtronic and as a speaker for AstraZeneca and Amgen. She has received grants from AstraZeneca, Novo Nordisk, Boehringer Ingelheim, Janssen and Sanofi‐Aventis and Eli Lilly. The other authors declare no conflict of interest.

## References

[1] Misra S , Ke C , Srinivasan S , et al. Current insights and emerging trends in early‐onset type 2 diabetes. Lancet Diabetes Endocrinol. 2023;11(10):768‐782. doi:10.1016/S2213-8587(23)00225-5 37708901

[2] Barker MM , Davies MJ , Sargeant JA , et al. Age at type 2 diabetes diagnosis and cause‐specific mortality: observational study of primary care patients in England. Diabetes Care. 2023;46(11):1965‐1972. doi:10.2337/dc23-0834 37625035

[3] National Diabetes Audit . NHS England Digital. Accessed October 3, 2024. https://digital.nhs.uk/data‐and‐information/publications/statistical/national‐diabetes‐audit

[4] Misra S , Holman N , Barron E , et al. Characteristics and care of young people with type 2 diabetes included in the national diabetes audit datasets for England. Diabet Med. 2023;40(1):e14940. doi:10.1111/dme.14940 36054265 PMC10087129

[5] NHS . England » NHS rolls out world‐first programme to transform diabetes care for under 40s. Accessed November 30, 2023. https://www.england.nhs.uk/2023/08/nhs‐rolls‐out‐world‐first‐programme‐to‐transform‐diabetes‐care‐for‐under‐40s/

[6] Ke C , Stukel TA , Shah BR , et al. Age at diagnosis, glycemic trajectories, and responses to oral glucose‐lowering drugs in type 2 diabetes in Hong Kong: a population‐based observational study. PLoS Med. 2020;17(9):e1003316. doi:10.1371/journal.pmed.1003316 32946450 PMC7500681

[7] Barker MM , Zaccardi F , Brady EM , et al. Age at diagnosis of type 2 diabetes and cardiovascular risk factor profile: a pooled analysis. WJD. 2022;13(3):260‐271. doi:10.4239/wjd.v13.i3.260 35432761 PMC8984563

[8] Nanayakkara N , Curtis AJ , Heritier S , et al. Impact of age at type 2 diabetes mellitus diagnosis on mortality and vascular complications: systematic review and meta‐analyses. Diabetologia. 2021;64(2):275‐287. doi:10.1007/s00125-020-05319-w 33313987 PMC7801294

[9] Sargeant JA , Brady EM , Zaccardi F , et al. Adults with early‐onset type 2 diabetes (aged 18‐39 years) are severely underrepresented in diabetes clinical research trials. Diabetologia. 2020;63(8):1516‐1520. doi:10.1007/s00125-020-05174-9 32483683 PMC7351852

[10] Zeitler P , Galindo RJ , Davies MJ , et al. Early‐onset type 2 diabetes and Tirzepatide treatment: a post hoc analysis from the SURPASS clinical trial program. Diabetes Care. 2024;47(6):1056‐1064. doi:10.2337/dc23-2356 38639997 PMC11116907

[11] TODAY Study Group . Pregnancy outcomes in Young women with youth‐onset type 2 diabetes followed in the TODAY study. Diabetes Care. 2021;45(5):1038‐1045. doi:10.2337/dc21-1071 34880068 PMC9174960

[12] Murphy HR , Howgate C , O'Keefe J , et al. Characteristics and outcomes of pregnant women with type 1 or type 2 diabetes: a 5‐year national population‐based cohort study. Lancet Diabetes Endocrinol. 2021;9(3):153‐164. doi:10.1016/S2213-8587(20)30406-X 33516295

[13] Creţu D , Cernea S , Onea CR , Pop RM . Reproductive health in women with type 2 diabetes mellitus. Hormones (Athens). 2020;19(3):291‐300. doi:10.1007/s42000-020-00225-7 32613536

[14] Zhu XB , Niu ZH , Fan WM , Sheng CS , Chen Q . Type 2 diabetes mellitus and the risk of male infertility: a mendelian randomization study. Front Endocrinol (Lausanne). 2023;14:1279058. doi:10.3389/fendo.2023.1279058 38152129 PMC10752377

[15] Liu Q , Tang B , Zhu Z , et al. A genome‐wide cross‐trait analysis identifies shared loci and causal relationships of type 2 diabetes and glycaemic traits with polycystic ovary syndrome. Diabetologia. 2022;65(9):1483‐1494. doi:10.1007/s00125-022-05746-x 35771237 PMC9345824

[16] Wijayaratna S , Lee A , Park HY , et al. Socioeconomic status and risk factors for complications in young people with type 1 or type 2 diabetes: a cross‐sectional study. BMJ Open Diabetes Res Care. 2021;9(2):e002485. doi:10.1136/bmjdrc-2021-002485 PMC8719138 34969690

[17] TODAY Study Group , Bjornstad P , Drews KL , et al. Long‐term complications in youth‐onset type 2 diabetes. N Engl J Med. 2021;385(5):416‐426. doi:10.1056/NEJMoa2100165 34320286 PMC8697255

[18] Ke C , Lau E , Shah BR , et al. Excess burden of mental illness and hospitalization in Young‐onset type 2 diabetes: a population‐based cohort study. Ann Intern Med. 2019;170:145‐154. doi:10.7326/M18-1900 30641547

[19] Barker MM , Davies MJ , Zaccardi F , et al. Age at diagnosis of type 2 diabetes and depressive symptoms, diabetes‐specific distress, and self‐compassion. Diabetes Care. 2023;46(3):579‐586. doi:10.2337/dc22-1237 36630531 PMC10020022

[20] Hackett RA , Steptoe A . Type 2 diabetes mellitus and psychological stress ‐ a modifiable risk factor. Nat Rev Endocrinol. 2017;13(9):547‐560. doi:10.1038/nrendo.2017.64 28664919

[21] Bobo WV , Cooper WO , Stein CM , et al. Antipsychotics and the risk of type 2 diabetes mellitus in children and youth. JAMA Psychiatry. 2013;70(10):1067. doi:10.1001/jamapsychiatry.2013.2053 23965896

[22] Dziewa M , Bańka B , Herbet M , Piątkowska‐Chmiel I . Eating disorders and diabetes: facing the dual challenge. Nutrients. 2023;15(18):3955. doi:10.3390/nu15183955 37764739 PMC10538145

[23] Speight J , Holmes‐Truscott E . Challenging diabetes stigma starts and ends with all of us. The Lancet Diabetes & Endocrinology. 2023;11(6):380‐382. doi:10.1016/S2213-8587(23)00084-0 37080229

[24] Brouwer AM , Salamon KS , Olson KA , et al. Adolescents and type 2 diabetes mellitus: a qualitative analysis of the experience of social support. Clin Pediatr (Phila). 2012;51(12):1130‐1139. doi:10.1177/0009922812460914 23034947

[25] Wong SKW , Soon W , Griva K , Smith HE . Identifying barriers and facilitators to self care in young adults with type 2 diabetes. Diabet Med. 2024;41:e15229. doi:10.1111/dme.15229 37767739

[26] Croke S , Volkmann AM , Perry C , et al. What are the perspectives of adults aged 18–40 living with type 2 diabetes in urban settings towards barriers and opportunities for better health and well‐being: a mixed‐methods study. BMJ Open. 2023;13(9):e068765. doi:10.1136/bmjopen-2022-068765 PMC10514606 37730399

[27] Haines L , Wan KC , Lynn R , Barrett TG , Shield JPH . Rising incidence of type 2 diabetes in children in the U.K. Diabetes Care. 2007;30(5):1097‐1101. doi:10.2337/dc06-1813 17259470

[28] Singh H , Cinnirella M , Bradley C . Support systems for and barriers to diabetes management in south Asians and whites in the UK: qualitative study of patients' perspectives. BMJ Open. 2012;2(6):e001459. doi:10.1136/bmjopen-2012-001459 PMC3532968 23151392

[29] Misra S , Gable D , Khunti K , et al. Developing services to support the delivery of care to people with early‐onset type 2 diabetes. Diabet Med. 2022;39(10):e14927. doi:10.1111/dme.14927 35900910 PMC9542364

[30] Seidu S , Davies MJ , Mostafa S , de Lusignan S , Khunti K . Prevalence and characteristics in coding, classification and diagnosis of diabetes in primary care. Postgrad Med J. 2014;90(1059):13‐17. doi:10.1136/postgradmedj-2013-132068 24225940

[31] Stratton IM , Adler AI , Neil HA , et al. Association of glycaemia with macrovascular and microvascular complications of type 2 diabetes (UKPDS 35): prospective observational study. BMJ. 2000;321(7258):405‐412. doi:10.1136/bmj.321.7258.405 10938048 PMC27454

[32] Roland M , Guthrie B . Quality and outcomes framework: what have we learnt? BMJ. 2016;354:i4060. doi:10.1136/bmj.i4060 27492602 PMC4975019

[33] EDEN . EDEN. May 23, 2024. Accessed May 23, 2024. https://www.edendiabetes.com

[34] Dasgupta K , Boulé N , Henson J , et al. Remission of type 2 diabetes and improved diastolic function by combining structured exercise with meal replacement and food reintroduction among young adults: the RESET for REMISSION randomised controlled trial protocol. BMJ Open. 2022;12(9):e063888. doi:10.1136/bmjopen-2022-063888 PMC9494595 36130753

[35] Hadjiconstantinou M , Chauhan R , Arsenyadis F , et al. The M3 development group. P358 the development of an innovative multi‐factorial management intervention to address multi‐morbidity in early‐onset type 2 diabetes. Diabet Med. 2023;40(S1):e15048. doi:10.1111/dme.15048

